# Alisol A inhibits and stabilizes atherosclerotic plaques by protecting vascular endothelial cells

**DOI:** 10.3389/fphar.2024.1493948

**Published:** 2024-10-25

**Authors:** Yang Ma, Dingzhong Song, Jie Yuan, Wusi Hao, Jianqiang Xi, Chunping Yuan, Zhihong Cheng

**Affiliations:** ^1^ China State Institute of Pharmaceutical Industry, National Advanced Medical Engineering Research Center, Shanghai, China; ^2^ Shanghai Engineering Technology Research Center for Pharmaceutical Intelligent Equipment, Shanghai, China

**Keywords:** alisol A, atherosclerotic plaques, vascular endothelial cells, TNF pathway, molecular docking

## Abstract

**Background and aims:**

Dysfunction of endothelial cells represents a crucial aspect in the pathogenesis of atherosclerosis. The aim of this study was to explore the protective effects of alisol A on vascular endothelial cells and its possible mechanisms.

**Methods:**

An atherosclerosis model was established by feeding ApoE-/- mice with high-fat chow. Alisol A (150 mg/kg/d) or atorvastatin (15 mg/kg/d) was administered, and the levels of blood lipids were evaluated. The effect of the drugs on atherosclerotic plaques was observed by staining the aorta with Sudan IV. *In vitro* experiments were conducted using human aortic endothelial cells (HAECs) to assess the effects of alisol A on cell proliferation, migration, tubulation, secretion, and cellular integrity by CCK-8 assay, wound healing assay, angiogenesis assay, NO secretion, and release of LDH. Transcriptomics and molecular docking were used to explore the mechanism of plaque inhibition and stabilization by alisol A.

**Results:**

Alisol A significantly reduced the aortic plaque area in ApoE^−/−^ mice fed with high-fat chow. *In vitro*, alisol A had a protective effect on HAECs, which was reflected in the inhibition of vascular endothelial cell proliferation, promotion of NO secretion by vascular endothelial cells, inhibition of vascular endothelial cell migration and angiogenesis, and the maintenance of cell membrane integrity. Therefore, alisol A inhibited and stabilized atherosclerotic plaques and slowed down the process of atherosclerosis. Transcriptomics studies showed 4,086 differentially expressed genes (DEGs) in vascular endothelial cells after alisol A treatment. Enrichment analysis indicated that many genes involved in TNF signaling pathway were differentially expressed, and inflammatory genes were suppressed. The molecular docking results verified the hypothesis that alisol A has a low binding energy after docking with TNF target, and TNF could be a potential target of alisol A.

**Conclusion:**

Alisol A produced protection on vascular endothelial cells, achieving inhibition and stabilization of atherosclerotic plaques.

## Introduction

Atherosclerosis is a chronic inflammatory disease characterized by lipid deposition, plaque formation, arterial wall thickening and lumen stenosis. Atherosclerosis is caused by a variety of injuries, and it is the basis of many cardiovascular and cerebrovascular diseases (Falk, 2006).

**FIGURE 1:**
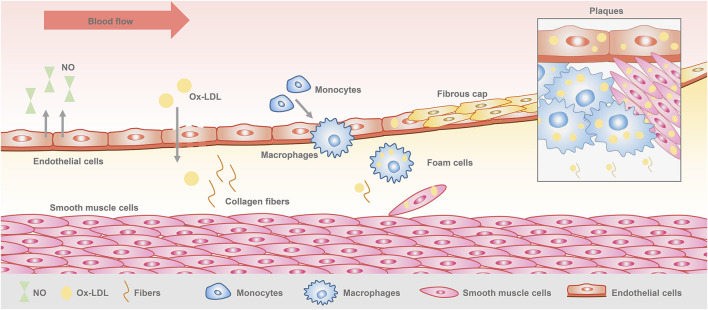
Pathogenesis of atherosclerosis.

Vascular endothelial cells are monolayer cells lining the surface of the vascular lumen, with tight connections between the cells, forming a barrier between blood and tissues (Dejana and Orsenigo, 2013). They are located in the innermost layer of blood vessels and capable of maintaining vascular homeostasis and regulating vascular function. Endothelial cells play an important role in the initiation and development of atherosclerosis (Komarova and Malik, 2010). When the vascular microenvironment is altered, physical and chemical factors stimulate the endothelial cells, resulting in endothelial dysfunction, which is considered as the beginning of atherosclerosis (Rajendran et al., 2013). Lipids accumulate through the damaged endothelial cells to the subendothelial membrane, complex sugars and fibrous matrix followed by continuous proliferation, localized large amounts of deposited calcium, causing inflammatory cell infiltration. Monocytes and smooth muscle cells convert to foam cells through phagocytosis of lipids, while the synthesis of extracellular matrix increases, and finally forming a space occupying lesion. At this time, plaque is formed (Wu et al., 2023) (Figure 1). Early plaques are relatively stable in a state of inflammatory cell infiltration. As the damage increases, the plaques develop towards poor stability and a tendency to form clots, and then creating vulnerable plaques (Weng et al., 2022). Multiple studies have shown that secondary thrombosis caused by rupture of vulnerable plaques is the fundamental cause of acute cardiovascular events (Virmani et al., 2000; Virmani et al., 2006). Therefore, controlling plaque formation and development is crucial for the treatment of atherosclerosis.

There has been a burgeoning emphasis on vascular endothelial cells, due to their distinctive positioning and functions in the formation of atherosclerosis. Currently, atherosclerosis is clinically treated with drugs, which mainly focus on the regulation of factors such as low density lipoprotein cholesterol (LDL-C), platelet aggregation, inflammatory factors, and blood pressure, i.e., by regulating the microenvironment of the vascular endothelial cells to control the development of plaques (Hetherington and Totary-Jain, 2022). There is no effective treatment for vascular endothelial cells themselves currently. Our research endeavors are captivated by endothelial cells, with the aspiration to facilitate plaque regression through targeted interventions on the vascular endothelial cell.

At present, statins are the first-line drugs for reducing blood lipids and anti atherosclerosis. However, they have the problem of intolerance for long-term use, which is prone to cause blood glucose rise, muscle soreness and occasional fatal side effects of rhabdomyolysis (Montastruc, 2023; Reith et al., 2024). With the understanding of the pathogenesis of atherosclerosis, great progress has been made in the treatment, especially the use traditional Chinese medicine (TCM) has been gradually accepted and widely used because of its obvious effect, less toxic and side effects. However, there are still some challenges, such as the lack of unified efficacy evaluation criteria and large sample double-blind research data, which limits the application of traditional Chinese medicine treatment methods (Liu et al., 2023).

With thousands of years of history of clinical use, traditional Chinese medicine Alismatis Rhizoma (Zexie) is extensively utilized for its diuretic properties and its ability to dispel dampness. It has garnered considerable attention in clinical settings due to its pronounced effects in reducing hyperlipidemia. Alisol A, the active triterpenoid component in Alismatis Rhizoma, has a significant inhibitory effect on atherosclerotic plaques.

Our research team has been dedicated to exploring natural products as therapeutic agents in the combat against atherosclerosis, and now we have successfully secured the clinical trial license for Zexie Jiangzhi Capsule from the China Food and Drug Administration, and completed the Phase I clinical study (Record number: CTR20212260, CTR20231110, CTR20232153). Based on the excellent therapeutic efficacy of Zexie Jiangzhi Capsule with alisol A as the main ingredient, we have confirmed that alisol A can inhibit macrophage foaming, reduce blood lipids in animals, and activate AMPK targets in the laboratory (Wang et al., 2020). To further explore the protective effects of alisol A on blood vessels, we established a vascular endothelial cell model in this study, and alisol A was directly applied to vascular endothelial cells, and changes in cell proliferation, migration, tube-forming, and secretion abilities were observed. It was found that alisol A had a positive effect on inhibiting and stabilizing atherosclerotic plaques. Then, we performed transcriptomic analysis and molecular docking validation to elucidate the possible mechanism of action of alisol A.

## Materials and methods

### Animals

Twenty four apolipoprotein E-deficient (ApoE^−/−^) male mice (approximately 8 weeks old) were purchased from GemPharmatech Co., Ltd. (Jiangsu, China). All mice were kept in the animal house of China State Institute of Pharmaceutical Industry, where they were maintained in a 12 h day/night cycle at a temperature of 20°C–22°C and relative humidity of 40%–70%, with free access to food and water *ad libitum*. All the experiment procedures were conducted in accordance to the guidelines of the Animal Ethics Committee of China State Institute of Pharmaceutical Industry to minimize the suffering of the animals.

All the mice were randomly divided into control group, model group, alisol A group (A) and Atorvastatin (AT) group (n = 6). The control group was fed with standardized chow (BEIJING HFK BIOSCIENCE CO., LTD., H10010, China), the model group was given high-fat chow (BEIJING HFK BIOSCIENCE CO., LTD., H10141, China), and A group was given high-fat chow mixed with alisol A (Self-made, dosage 150 mg/kg/d) in high-fat chow, and the AT group was given high-fat chow mixed with atorvastatin (dose 15 mg/kg/d). All groups of mice were fed for 16 weeks.

### Serum biochemistry assays

Serum was collected from each group of mice and the concentrations of total cholesterol (TC), triglycerides (TG), low-density lipoprotein cholesterol (LDL-C), and high density lipoprotein cholesterol (HDL-C) were measured according to the protocols of the kit (RECOM BIO, XZ017801, XZ027801, XZ037801, Nanjing Jiancheng Bioengineering Institute, A112-1-1,China), which was used to evaluate the levels of blood lipids.

### Sudan IV staining

Experimental animals were killed by cervical dislocation. The thoracic cavity of mice was rapidly opened, and the aorta of mice was obtained under stereomicroscope. The aortic arch and heart were taken and stored in 4% paraformaldehyde, stained with Sudan IV staining solution for 6 min, decolorized in 80% ethanol for 3 min, finally washed in PBS for 2 min. The aortic vessels were fixed with a small needle to spread out. The atheromatous plaques in the aortic arch and bifurcation were photographed under a stereomicroscope. The images were stitched together to form a complete picture by Photoshop software, and the ratio of the plaque area to total vessel area was calculated by Image Pro software.

### Cell culture

Human aortic endothelial cells (HAECs) were purchased from YUCHI BIOLOGY (Shanghai, China). The cells were cultured in endothelial cell medium (ECM, ScienCell, 1001, United States),supplemented with 5% fetal bovine serum (FBS, ScienCell, 0025, United States), 1% dual anti-penicillin/streptomycin (P/S, ScienCell, 0503, United States) and 1% growth supplementation factor (ECGS, ScienCell, 1052, United States), at 37°C and 5% CO_2_ atmosphere.

### Cell proliferation assay

The cell viability was detected by CCK8 method. HAEC cells in logarithmic growth phase (no more than 10 passages) were inoculated into 96-well cell culture plates, given medium containing different concentrations of alisol A for incubating 24 h, 48 h and 72 h, and then operated according to the protocols of the kit (Cell Counting Kit-8, Beyotime, C0038, China). Light absorbance was measured at 450 nm wavelength and the cell viability was assessed.

### Evaluation of cell migration

The migration characteristics were evaluated using the scratch assay. HAEC cells in logarithmic growth phase (no more than 10 passages) were inoculated into 24-well cell culture plates, and scratched vertically with a 200 μL pipette tip after 80% cell fusion. Then, cells were incubated in medium (serum-free treatment) containing different concentrations of alisol A for 8 h. The cells were observed and photographed under a microscope, and the ratio of migratory areas and distances were calculated by ImageJ software.

### Angiogenesis experiment

HAEC cells in logarithmic growth phase (no more than 10 passages) were inoculated into 96-well cell culture plates covered with Matrix-Gel™ (Beyotime, C0372, China), and given medium (serum-free treatment) containing different concentrations of alisol A for incubating 6 h. The cells were observed and photographed microscopically, and the number of junctions and the total length of the tubes were analyzed and compared with each other by using the Angio Tool software, to evaluate the angiogenesis.

### Measurement of nitric oxide determination

The Griess method was used to determine the secretion of NO. HAEC cells in logarithmic growth phase (no more than 10 passages) were inoculated into 96-well cell culture plates, and then divided into control group, high-fat stimulation group (FFA, 200 μM), and different concentration of alisol A treated group for incubation for 48 h. The cell culture medium was measured according to the protocols of the kit (Total Nitric Oxide Assay Kit, Beyotime, S0021S, China). The absorbance was measured at 545 nm, and the NO concentration was calculated according to the standard curve.

### Detection of lactate dehydrogenase (LDH) release

Cellular release of LDH was measured using a colorimetric assay to evaluate the integrity of the cell membrane. HAEC cells in logarithmic growth phase (no more than 10 passages) were inoculated into 96-well cell culture plates, divided into control group, oxidative damage group (hydrogen peroxide, 100 μM), and different concentration of alisol A treated groups for incubation for 48 h. The cell culture medium was measured according to the protocols of the kit (LDH Cytotoxicity Assay Kit, Beyotime, C0017, China), and the absorbance were measured at 492 nm to calculate the LDH release rate.

### Transcriptome analysis

Human microvascular endothelial cells (HMEC-1) were inoculated in 60 mm culture dishes, and divided into control group, inflammation injury group (LPS, 1 μg/ml) and alisol A treatment group. In the A group, the cells were treated with alisol A (10 μM) for 72 h, then LPS was added, and after 6 h of stimulation, the cells were scraped off with a spatula and collected. Then the total RNA was extracted with TRIzol reagent (Beyotime, R0016, China). Among them, the control group was labeled KB, the A group was labeled A, and the model group was labeled Model.

The concentration and purity of the extracted RNA were examined by Nanodrop (2000). RNA completeness was detected by agarose gel electrophoresis, and RNA quality number (RQN) was determined by Agilent 5,300. The following requirements should be met: the amount of RNA should be greater than 1 μg; the concentration of RNA should be higher than 30 ng/μL; RQN should greater than 6.5; the ratio of OD260/280 should be between 1.8 and 2.2.

Eukaryotic mRNA sequencing was performed on a NovaSeq X Plus platform. Fastp quality control and RSEM software were used for expression analysis, and DESeq2 software for differential expression analysis. Enrichment analysis was performed by Goatools and Python scipy software, using the Fisher’s exact test.

### Molecular docking

To evaluate the binding energy and interaction mode between alisol A and its targets, the 3D structures of key targets were downloaded from the PDB database (https://www.rcsb.org/) and the molecular structure of alisol A was obtained from the PubChem compound database (https://pubchem.ncbi.nlm.nih.gov/). The target protein and alisol A were dehydrogenated and hydrogenated, and the docking pocket was set as a square of 30 A × 30 A × 30 A with a lattice distance of 0.05 nm. Molecular docking was fitted by using AutodockVina 1.2.2 software, and the binding energies were calculated, finally PyMOL software was used for visualization.

### Statistical analysis

Each experiment was repeated at least three times. Data were expressed as mean ± standard deviation. One-way analysis of variance (ANOVA) was used for multiple group comparisons, and t-test was used for two-group comparisons. Bar graphs were plotted using GraphPad Prism 8.0 software, while *p* < 0.05 was considered statistically significant.

## Results

### Alisol A inhibited atherosclerotic plaque *in vivo*

**FIGURE 2:**
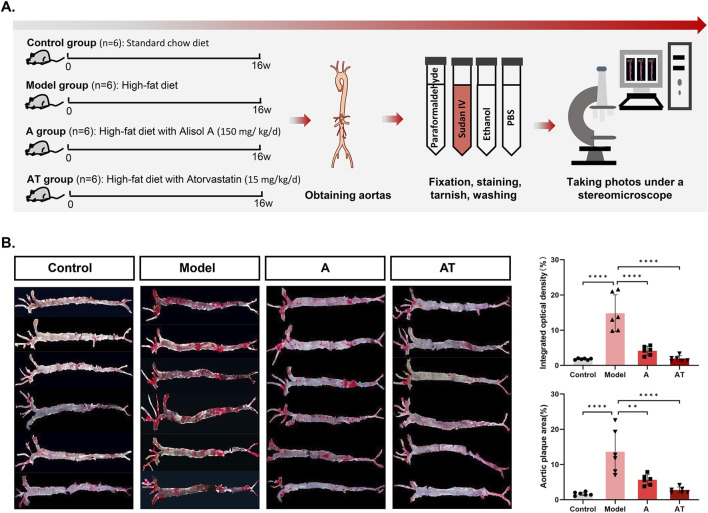
Alisol A inhibited atherosclerotic plaque *in vivo*. **(A)** Flowchat of animal experiment. **(B)** Sudan IV staining of the entire aorta to assess atherosclerotic plaque load of mice and the quantification of the plaque density by optical techniques to calculate the gross plaque areas in the entire aorta (n = 6).

Sudan IV staining demonstrated obvious lipid deposition in arterial lumen and severe atherosclerotic plaques in the model group. Plaque staining area was significantly reduced after alisol A treatment. Quantitative analysis showed that plaque area significantly increased from (1.68 ± 0.49)% to (13.62 ± 6.18)% in the model group after high-fat feed feeding (****P < 0.0001), and the plaque area was significantly reduced to (5.66 ± 1.50)% compared with the model group after alisol A treatment (**P < 0.01) (Figure 2B).

### Alisol A had no significant effect on lipid levels in ApoE^−/−^ mice

There were no significant differences in serum concentrations of total cholesterol (TC), triglycerides (TG), low-density lipoprotein cholesterol (LDL-C), and highdensity lipoprotein cholesterol (HDL-C) between groups of experimental animals (data not shown).

### Alisol A inhibited cell viability

**FIGURE 3:**
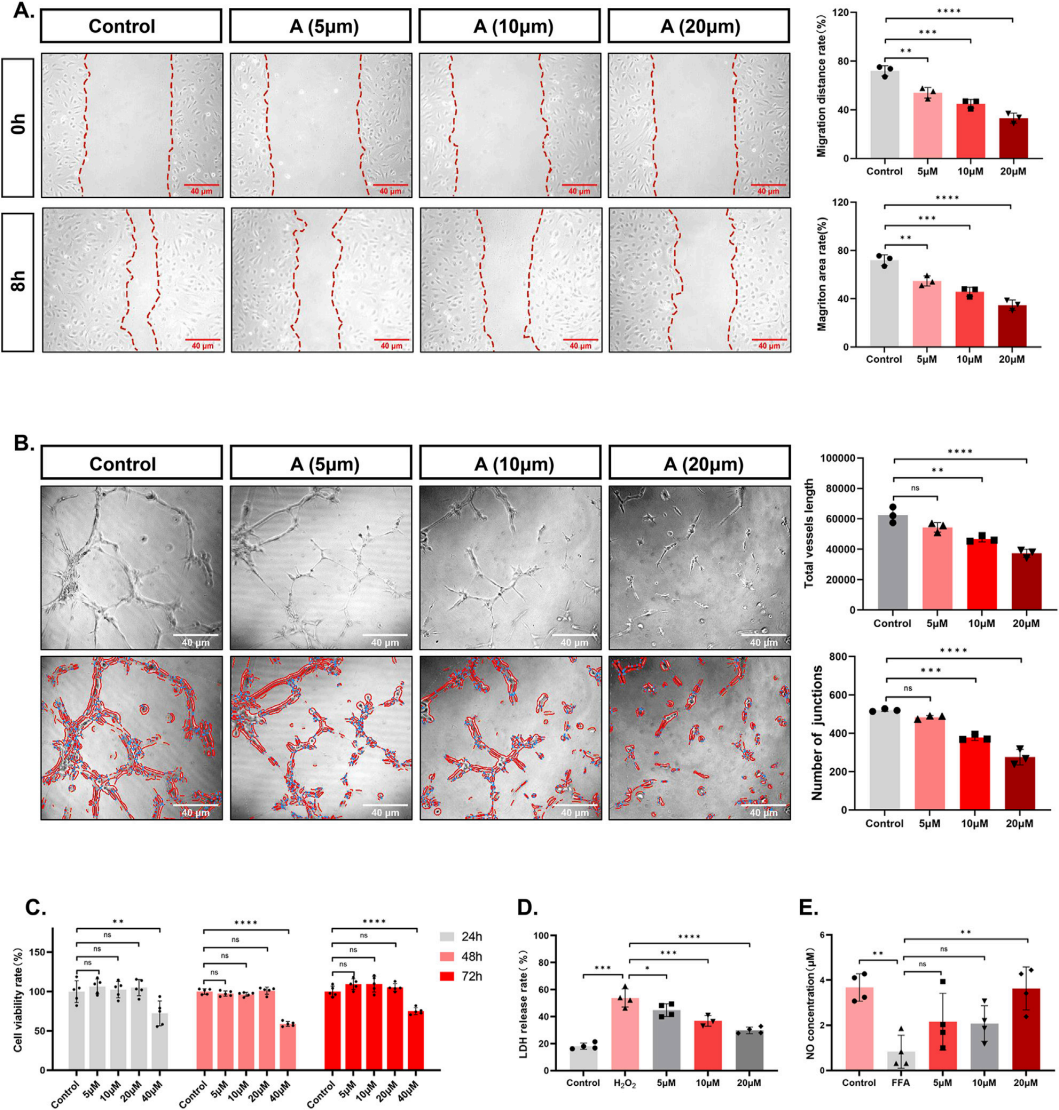
The effects of alisol A on the proliferation, migration, angiogenesis, secretion, and integrity of vascular endothelial cells. **(A)** Representative pictures and quantitative estimation of the migration by wound healing analysis (n = 3). **(B)** Representative pictures and quantitative estimation of the tube formation assay (n = 3). **(C)** The viability of HAECs and quantitative measurement of the CCK-8 assay (n = 5). **(D)** Quantitative analysis of the LDH release rate (n = 3,4). **(E)** Quantitative analysis of the NO concentration (n = 4).

Cell proliferation is one of the important physiological functions of living cells, and cell viability can indirectly reflect cell proliferation (Khalef et al., 2024). Taking 24 h as an example, the cell viability of control group was (99.80 ± 13.87)%, and there was no significant change in cell viability after incubating with low concentration of alisol A. When the concentration was increased up to 40 μM, the cell viability decreased to (72.24 ± 15.68)% (control group vs. 40 μM group, **P < 0.01), indicating significant inhibition of cell viability (Figure 3C). The change trends of cell viability at 48 h and 72 h were consistent results. It can be inferred that alisol A has an inhibitory effect on the proliferation of vascular endothelial cells, and its inhibitory effect is related to the concentration of alisol A (when the concentration reaches 40 μM, it shows a significant decrease), and is not related to the incubation time.

### Alisol A inhibited cell migration

Mobility is a property of vascular endothelial cells, and wound healing experiments are often used to evaluate the migratory ability of cells (Montoro-García et al., 2023). As shown in the figure, the percentage of migratory distance in control group was (71.97 ± 4.24)% after 8 h. However, the presence of alisol A decreased the percentage of migratory distance of the cells, and the percentage of migratory distance was further decreased with the increase of concentration to (53.97 ± 4.37)%, (44.92 ± 3.53)%, and (33.08 ± 4.11)% (control group vs. 5 μM group **P < 0.01, control group vs. 10 μM group ***P < 0.001, control group vs. 20 μM group ****P < 0.0001). The trend of migration area is consistent with the distance (Figure 3A).

### Alisol A inhibited angiogenesis

In the physiological state, vascular endothelial cells exhibit angiogenesis properties (Kelley et al., 2022). After inoculation of low density of cells, the morphology of the cells was observed, and the number of bifurcation points and the length of the total tube formation were measured to evaluate the status of angiogenesis. As shown in Figure 3B, the formation of reticular vascular morphology in the field of view was inhibited after 6 h of alisol A treatment. The statistics showed that with the increase of the concentration of alisol A, the length of the total tube was further reduced from (62405.26 ± 5214.90), to (54307.70 ± 3138.88), (46699.08 ± 1953.67), and (37266.45 ± 2663.71) (control group vs. 10 μM group, **P < 0.01, control group vs. 20 μM group ****P < 0.0001). The number of junctions decreased from (520.33 ± 7.09) to (483.00 ± 9.54), (377.67 ± 15.04) and (275.33 ± 40.50) (control group vs. 10 μM group, ***P < 0.001, control group vs. 20 μM group, ****P < 0.0001). It can be inferred that slisol A has the effect of inhibiting angiogenesis.

### Alisol A promoted the secretion of NO

Endothelial cells have a secretory function, among which NO is an endothelial factor that is secreted outside to exert vasoprotective effects (Jiang et al., 2021). The concentration of NO in the culture medium can be used as an indicator to evaluate the secretory function of cells (Jiang et al., 2021). As can be seen from Figure 3E, FFA treatment caused the NO concentration in the cell culture medium to decrease from (3.67 ± 0.61) μM to (0.83 ± 0.73) μM. (control group vs. FFA group, **P < 0.01). However, there was a tendency for NO concentration to increase in the presence of alisol A. The concentration of NO in the presence of alisol A tended to increase. When the concentration of alisol A reached 20 μM, the NO concentration increased to (3.63 ± 0.95) μM, which was significantly different compared with the FFA group (FFA group vs. 20 μM group, **P < 0.01). These data suggest that alisol A has a restorative effect on decreasing the NO secretion caused by FFA.

### Alisol A reduced the release of LDH

When the cell membrane structure is damaged, intracellular lactate dehydrogenase is released into the extracellular space, so LDH release is viewed as an important indicator of the integrity of the cell membrane and is widely used in cytotoxicity assays (Kuma et al., 2018). Hydrogen peroxide treatment caused cellular LDH release to increase from (18.06 ± 2.33)% to (53.79 ± 6.74)% (control group vs. H_2_O_2_ group, ***P < 0.001). However, the LDH release rate tended to decrease in the presence of alisol A. The LDH release rate further decreased to (44.75 ± 4.71)%, (36.81 ± 3.93), even (29.78 ± 2.43)% with increasing concentration (H_2_O_2_ group vs. 5 μM group, *P < 0.05, H_2_O_2_ group vs. 10 μM group, ***P < 0.001, H_2_O_2_ group vs. 20 μM group ****P< 0.0001) (Figure 3D). These data suggest that alisol A has a protective effect against cell membrane breakdown brought about by hydrogen peroxide.

### Transcriptome analysis

**FIGURE 4:**
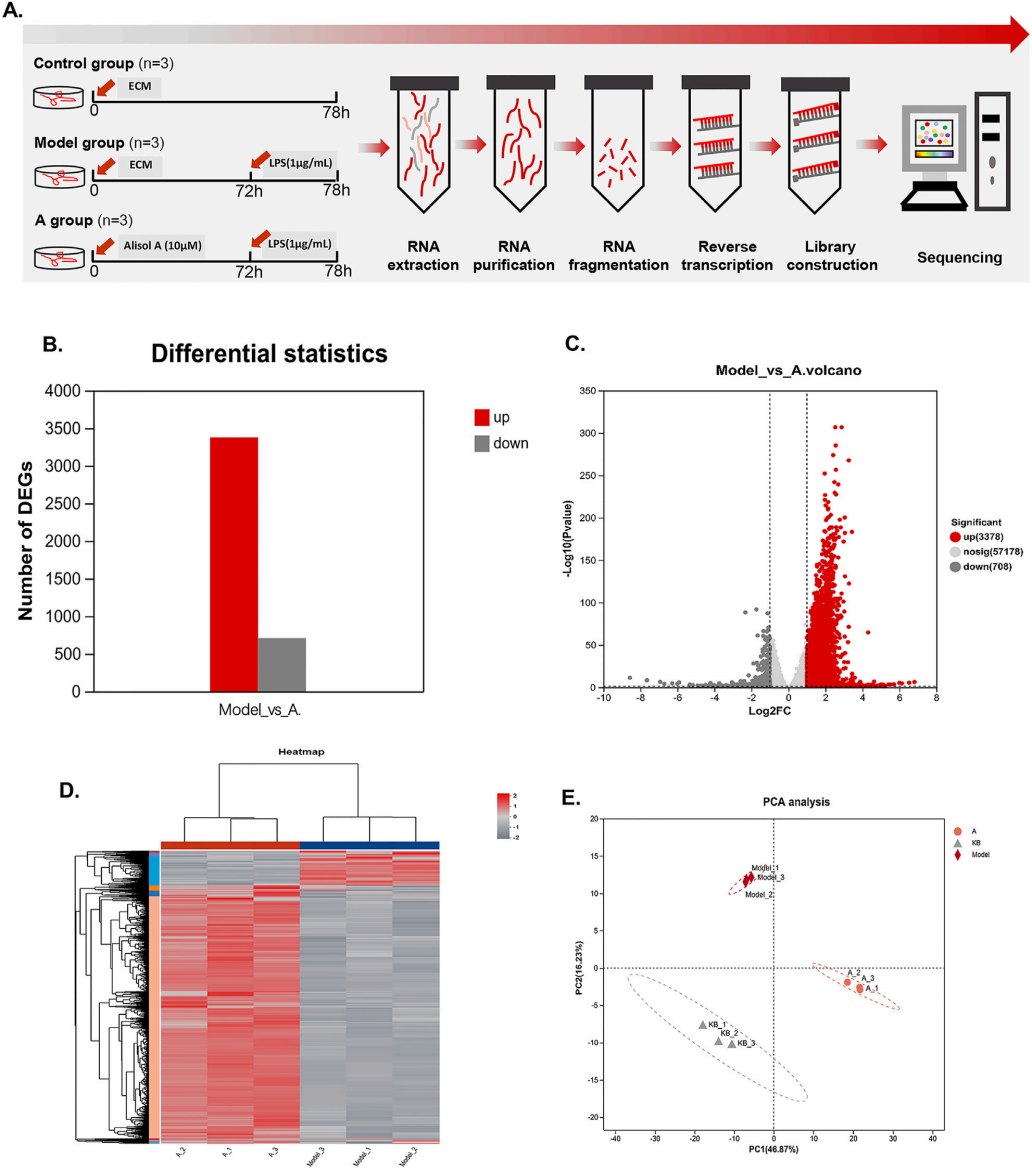
Transcriptional changes in HMEC-1. **(A)**Flowchart of transcriptome experiment. **(B)** Column graphs showed the quantity of DEGs. **(C)**Volcano plot showed the distribution of DEGs between the model group and alisol A group. Downregulation was depicted in dark grey, while upregulation was depicted in red. alisol A, 10 μM. **(D)** Heatmap showed the cluster of differential genes in alisol A treatment and model group. Up and downregulation were represented in red and grey. **(E)** PCA analysis showed differences intra group and inter group.

To explore the effects of alisol A on cell proliferation, migration, and tube-forming properties, we performed transcriptome analysis of vascular endothelial cells in the medium-dose group. Based on the expression quantification results, differentially expressed genes (DEGs) analysis was performed using DESeq2, screening for up and downregulated multiples greater than 2, *p*-value less than 0.05, with a total of 4,086 DEGs, of which 3,378 were upregulated (red) and 708 were downregulated (gray) (Figure 4B). The DEGs distribution can be represented by the volcano diagram (Figure 4C). The closer the points to the two sides and the upper side, the more significant the drug modulation effect. In the clustering heatmap, we can observe the expression value of each gene in different samples (red represents higher expression, while gray represents lower expression), and the genes clustered significantly under the effect of alisol A (Figure 4D). The PCA analysis showed good parallelism of biological replicates between samples within a group, and significant differences in data between groups (Figure 4D).

**FIGURE 5:**
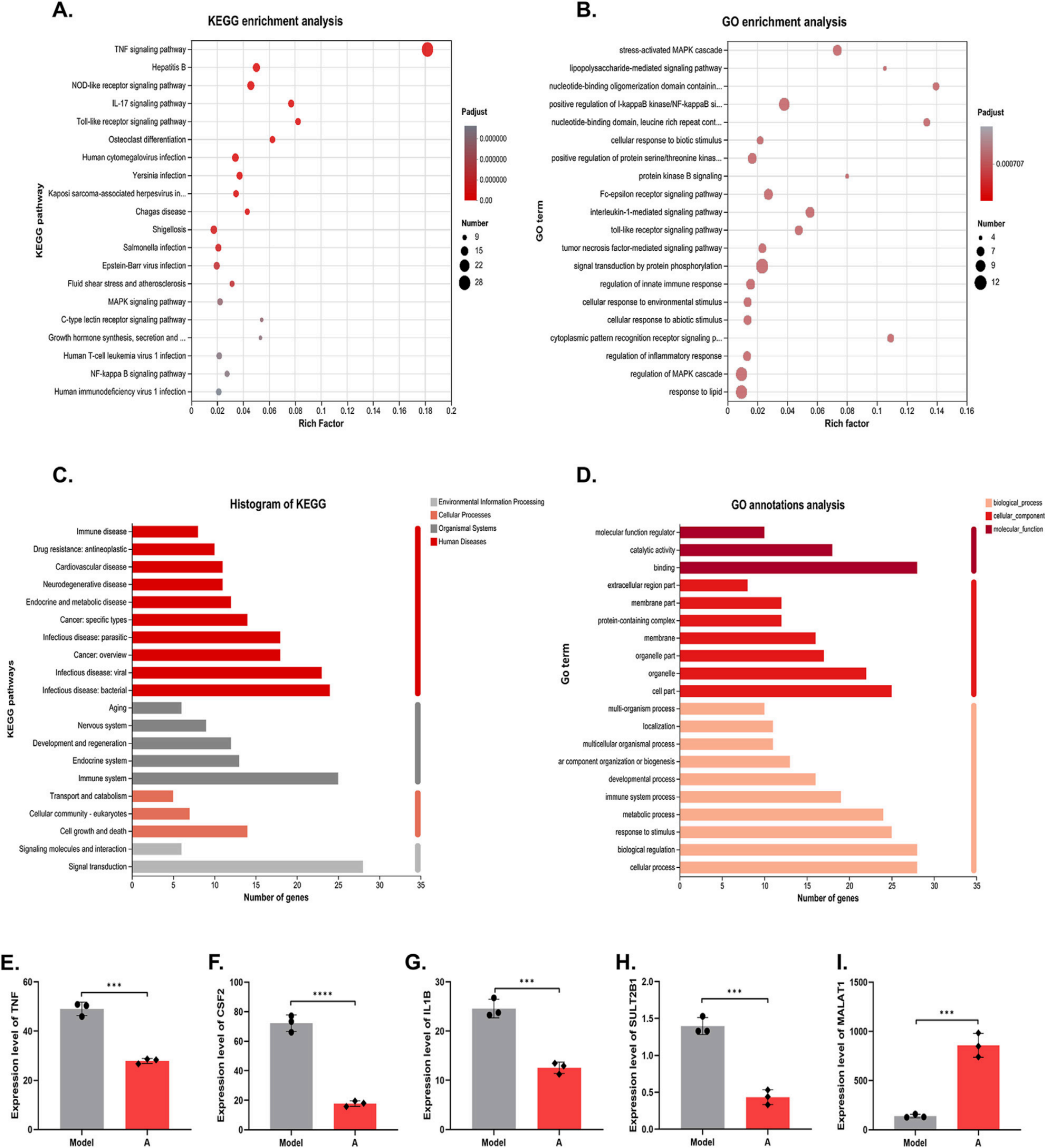
KEGG and GO analysis on the DEGs. **(A)** KEGG enrichment analysis. **(B)** GO enrichment analysis. **(C)** KEGG annotations analysis. **(D)** GO annotations analysis. **(E–I)** The mRNA level of TNF, CSF2, IL1B, SULT2B1 and MALAT1 (n = 3).

Functional enrichment analysis was performed on the genes to obtain the main functions of the genes in the set. KEGG enrichment analysis categorized and enriched the genes from the perspective of signaling pathways (Kanehisa et al., 2017). Figure 5A lists the top 20 pathways, of which the highest number of enriched genes was TNF pathway, suggesting that alisol A may intervene in plaque progression by modulating the effects of the TNF pathway. GO enrichment analysis encompasses three ontologies, which describe the molecular function, cellular components, and biological processes involved in genes (Mo et al., 2023). The result was shown in Figure 5B.

The enriched genes were further annotated and analyzed, i.e., genomic information and functional information were integrated, and the genes were classified according to signaling pathways or biological functions. The results showed that alisol A plays an important role in cell growth and apoptosis, immune system diseases, cardiovascular diseases, infectious diseases, and other processes. Further indications suggest that alisol A may intervene in the development of plaques through anti-inflammatory and other effects (Figures 5C, D).

At the micro level, we focused on five genes, TNF, CSF2, IL1B, SULT2B1 and MALAT1. TNF, CSF, and IL-1β are cytokines with pro-inflammatory effects, which act on vascular endothelial cells to promote blood vessel formation and cell proliferation (Zhou et al., 2020; Zhou et al., 2021). Alisol A showed a significant inhibitory effect on these genes. The results suggested that the alisol A exerted its inhibitory effect on vascular endothelial cell proliferation and tube formation by inhibiting the expression of the genes for TNF, CSF, and IL-1β. (Figures 5E–G). In addition, SULT2B1 and MALAT1 have been extensively studied in the field of cancer in recent years. Hangyu Pan found that inhibition of SULT2B1, reduced macrophage inflammatory response, stabilized plaque, and delayed the progression of atherosclerosis (Yin and Chen, 2020). Katharina M found that silencing of MALAT1 promotes endothelial cell migration, which indicated that MALAT1 inhibited endothelial cell migration (Michalik et al., 2014). The expression of MALAT1 was upregulated and that of SULT2B1 was downregulated in endothelial cells following incubation with alisol A (Figures 5H, I). The synergistic effect of them exerted the effect of inhibiting endothelial cell migration.

### Molecular docking validated the targets

**FIGURE 6:**
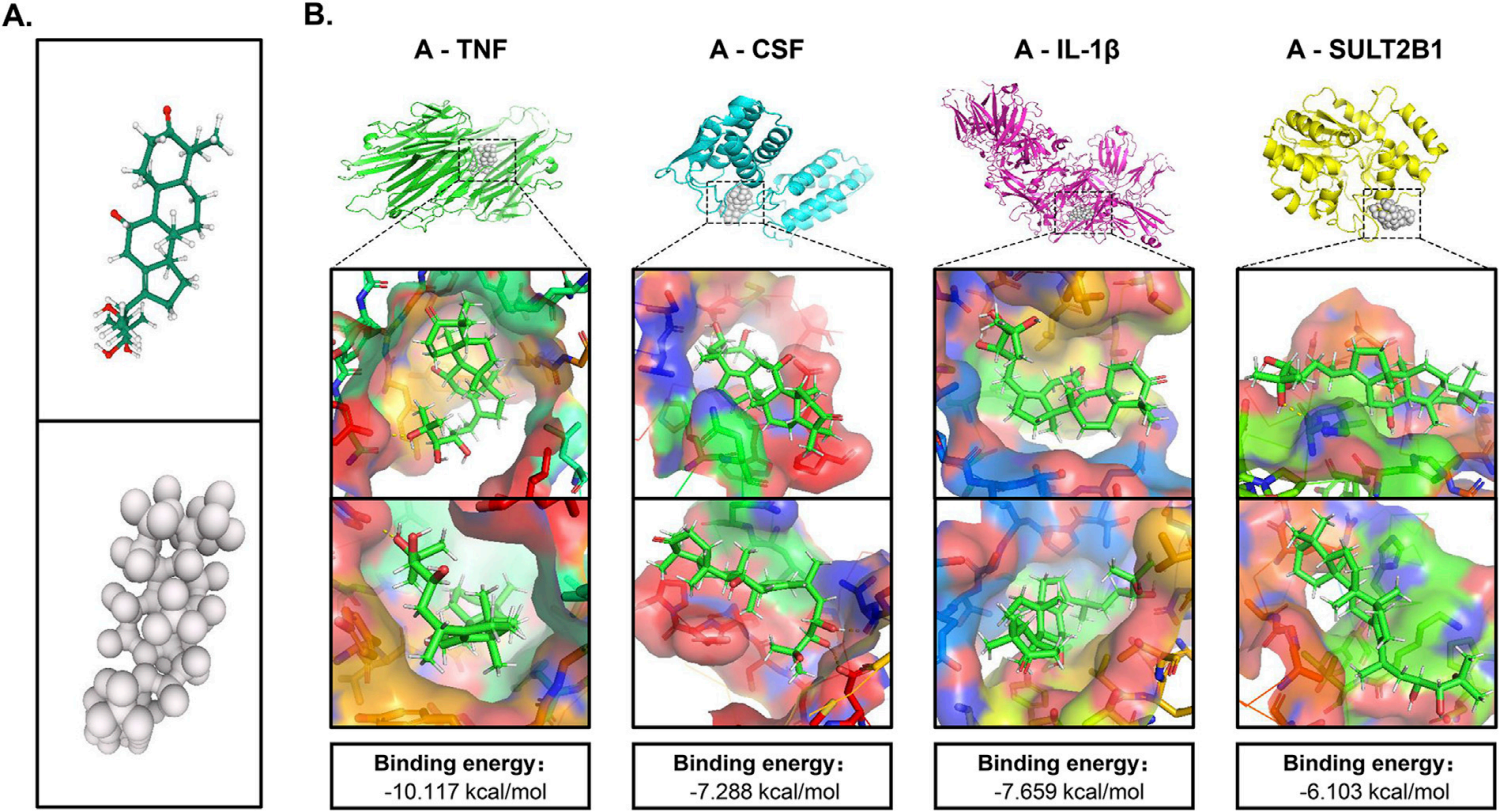
Molecular docking of crucial targets. **(A)** 3D structure of alisol A. **(B)** Binding mode and energy of alisol A and different targets.

To validate the transcriptomics findings, we performed molecular docking analysis on the targets TNF, CSF, IL-1β and SULT2B1 (MALAT1 is a noncoding gene and does not encode proteins) and assessed the effects of the alisol A on the above targets (Figure 6B). When the ligand binds to the target to form a conformationally stable structure, the lower the energy, the more stable the structure. When the binding energy is less than 0 kcal/mol, the molecular ligand can spontaneously bind to the protein receptor. If the binding energy is less than −5 kcal/mol, it indicates that their binding ability is stronger and the possibility of interaction is higher (Hsin et al., 2013). According to Figure 6B, alisol A has a low binding energy with all four targets, and has the lowest binding energy of −10.117 kcal/mol with TNF, indicating the best stability. TNF stimulates the cellular production of IL-1β, and it also enhances the CSF production, according to the docking results, alisol A can directly bind to and inhibit TNF, and then inhibit its downstream factors CSF and IL-1β production; it can also directly act on CSF and IL-1β to play an inhibitory role.

## Discussion

Vascular endothelial cells, smooth muscle cells and macrophages are collectively involved in atherosclerotic plaque formation (Bloom et al., 2023). Endothelial cells have many characteristics, such as secretion, proliferation, migration, and angiogenesis, all of which are important for the occurrence and development of atherosclerotic plaque. Damage to the vascular endothelium is often the initial stage of atherosclerosis. Maintaining the integrity of the endothelial cell membrane is of great significance in the early stages of plaque formation (Goossens, 2017).

Cell proliferation is an important physiologic function of living cells. Under certain stimulus conditions, vascular endothelial cells over proliferate, and the excess endothelial cells can move with lipids and participate in the formation of plaque (Bolatai et al., 2022). Therefore, inhibiting the proliferation of vascular endothelial cells, to a certain extent, can inhibit early plaque formation, and impede the development of already formed plaques.

Migration is a fundamental function of vascular endothelial cells, including four processes: extending pseudopods, constructing new adhesions, regulating cytosolic contraction and forward movement and tail retraction. The migration and movement of vascular endothelial cells bring about plaque instability (Chung et al., 2011). Therefore, inhibiting their migration has a stabilizing effect on plaques.

Immunohistochemical studies have found that plaques stability is closely related to the formation of neovascularization within the plaques (Vergallo and Crea, 2020). Neovascularization in physiological state generally exists between the outer and middle membranes of the canal wall and does not extend to the inner membrane. However, factors such as inflammatory reactions, elevated lipids and oxidative stress stimulate the growth of neovascularization toward the intima, thus affecting plaque stability (Takaya et al., 2006). In addition, neovascularization within plaques can induce plaque rupture and hemorrhage, promoting the development of stable plaques towards unstable plaques. As the number of neovessels increases, the clinical symptoms gradually worsen (Kelley et al., 2022).

Vascular endothelial cells can secrete NO to regulate the structure and function of blood vessels. NO can dilate blood vessels, regulate vascular tone, and alleviate luminal stenosis due to plaque development; NO can also resist platelet aggregation and reduce thrombotic events triggered by plaque dislodgement; and NO can improve the endothelial dysfunction, which plays a certain role in the initiation of the atherosclerotic plaque formation (Tousoulis et al., 2014; Förstermann et al., 2017; Yan et al., 2017). Therefore, the increased secretion of NO by vascular endothelial cells helps to impede plaque formation and development which bring about a positive effect on atherosclerosis.

The TNF signaling pathway is a complex molecular network involving interactions between multiple protein molecules. These molecules play different roles in the pathway and together form the molecular basis of the TNF signaling pathway. TNF is a multifunctional pro-inflammatory cytokine that binds to its receptors TNFR1 and TNFR2 and triggers signal transduction processes. It can activate the NF-κB signaling pathway to promote inflammatory response. TNF can also activate the mitogen activated protein kinase (MAPKs) pathway to enhance inflammatory response (van Loo and Bertrand, 2023). The TNF signaling pathway is closely related to cell proliferation, migration, and tube forming ability (Tian et al., 2015). For example, TNF can activate the downstream Wnt/β-catenin signaling pathway, accelerating cell proliferation (Zhao et al., 2020). TNF promotes cell migration through the ERK pathway. TNF can also promote the formation of new blood vessels by upregulating the expression of factors such as VEGF. In addition, TNF works synergistically with IL1β and IL6 to promote angiogenesis. Alisol A can bind to and inhibit TNF, thereby exerting cytoprotective effect and alleviating atherosclerosis. The specific downstream signals of TNF need further research.

The results of our study showed that alisol A inhibited plaque formation, protected the membrane integrity of vascular endothelial cells, inhibited endothelial cell proliferation, migration and angiogenesis, and also promoted the secretion of NO and therefore delayed the occurrence and development of atherosclerotic plaques, as well as brought about a positive effect on the stabilization of vulnerable plaque. Transcriptomics and molecular docking results showed that the mechanism by which alisol A exerted these effects was by inhibiting the inflammatory response, realizing the inhibition of the proliferation, migration, tube-forming and secretion properties of vascular endothelial cells, thus realizing the inhibition and stabilization of plaque, and slowing down the process of atherosclerosis. Alisol A had almost no significant effect on lipid levels in ApoE^−/−^ mice, it indicated that the inhibitory effect of alisol A on plaque is independent of the lipid-lowering effect (Ding et al., 2019).

In the previous study, our team had found that alisol A can play a lipid-lowering and anti-atherosclerotic role by promoting AMPK phosphorylation. While this study is the first time to show that alisol A acts directly on vascular endothelial cells, and found that alisol A has a vascular protective effect independent of its lipid-lowering effect, which further our understanding of the role of alisol A in atherosclerotic diseases and supported the possibility that it can exert its anti-atherosclerotic effect through multiple targets. The findings of this study enriched the research achievements in the field of traditional Chinese medicine treatment of atherosclerosis, and provided a new scheme for traditional Chinese medicine treatment of atherosclerosis. Based on the protective effect of alisol A on vascular endothelial cells, research and application can be expanded in hypertension, diabetes, Kawasaki disease and other diseases in the future. In addition, its application can be expand from cardiovascular disease to cerebrovascular disease.

## Data Availability

The raw data supporting the conclusions of this article will be made available by the authors, without undue reservation.
